# Gossypol-Induced Differentiation in Human Leukemia HL-60 Cells

**Published:** 2006-12

**Authors:** Wen-Qing Wang, Rong Li, Qing-Xian Bai, Yu-Hong Liu, Wei-Ping Zhang, Juan-Hong Wang, Zhe Wang, Yuan-Fei Li, Xie-Qun Chen, Gao-Sheng Huang

**Affiliations:** 1 *State Key Laboratory of Cancer Biology, Department of Pathology, Xijing Hospital, the Fourth Military Medical University, Xi’an, Shaanxi Provience, P. R. China;*; 2 *Department of Hematology, Xijing Hospital, the Fourth Military Medical University, Xi’an, Shaanxi Provience, P. R. China*

**Keywords:** gossypol, differentiation, leukemia, drug effects

## Abstract

The main treatment of leukemia is traditional radiochemotherapy, which is associated with serious side effects. In the past twenty years, differentiation was found as an important effective measure to treat leukemia with fewer side effects. Gossypol, a natural compound which has been used as an effective contraceptive drug, has been proposed to be a potent drug to treat leukemia, but the differentiation effect has not been studied. In the present study, we investigated the pro-differentiated effects, in vitro, of gossypol on the classic human myeloid leukemia HL-60 cell line. The effects of gossypol were investigated by using morphological changes, nitroblue tetrazolium (NBT) reduction, surface markers, cell-cycle analysis and Western blot analysis, etc. When HL-60 cells were incubated with low concentrations of gossypol (2-5μM) for 48hr, a prominent G0/G1 arrest was observed. At 96 hr of treatment, 90% of HL-60 cells differentiated, as evidenced by morphological changes, NBT reduction, and increase in cell surface expression of some molecules were detected. This study is the first to identify gossypol’s pro-differentiated effects on the leukemia cell line, and it induced differentiation through the PBK (PDZ-binding kinase)/TOPK (T-LAKcell-originated protein kinase) (PBK/TOPK) pathway. It is concluded that gossypol could induce differentiation in the leukemia HL-60 cells, and it may be a potential therapeutic agent, chemoprevention or chemotherapeutic adjuvant especially in combination drug therapy for leukemia.

## INTRODUCTION

Leukemia is a disease manifested by the failure of cell death, or inability of hematopoietic cells to differentiate into functional mature cells. The chemotherapy, which is cytotoxic to malignant clones, and cytodestructive to normal cells, is the conventional approach to treat leukemia. In these days, induction of differentiation or cell death in immature hematopoietic cells has been applied for leukemia prevention or therapy (1). Differentiation therapy, which is associated with less adverse effects, has been tested as a leukemia treatment modality. Differentiation therapy has been successful for acute myeloid leukemia (AML) (2). Several compounds including dimethyl sulfoxide, retinoic acid, phorbol ester and 1,25-dihydroxy vitamin D3 induce AML cells to differentiate toward mature cells. Among them, retinoic acid induces AML cells to differentiate toward granulocytes, whereas 1,25-dihydroxy vitamin D3 induces AML cells to differentiate toward monocytes (3). However, effective doses of these drugs in clinical studies produced objectionable side effects, physiological toxicity and drug resistance (4). It is very necessary for us to search for some new drugs to use in leukemia differentiation therapy.

Recently, more attention has been given in identifying naturally occurring cancer preventive or chemotherapeutic agents. Many phenolic and flavonoid compounds from plants have been shown to induce differentiation or apoptosis in human myelocytic leukemia cell lines (5). Gossypol [2,2-bi (8-formyl-1,6,7-trihydroxy-5-isopropyl-3- methylnaphthalene)] is a polyphenolic compound naturally occurring in pigment glands of cottonseed (gossypium) plants (6). It is originally identified as a male antifertility agent and has been used as an effective male contraceptive drug for many years (7). It has also been used in the treatment of endometriosis and uterine myoma, with some benefits noted also with cancer patients, suggesting it maybe an effective anti-solid tumor drug with fewer side effects (8, 9). Others have reported that gossypol demonstrated activity against cell lines resistant to anticancer agents such as adriamycin, vinblastin, and cisplatin (10). At the same time, oral administration of gossypol can be given for 6 months or more (11), providing more stable blood drug levels for leukemia therapy and for longer periods of time compared to other chemotherapeutic drugs. Though there are some side effects in gossypol, such as hypokalemia, reversible liver damage, and spermatogenic epithelium injury, they appear much simpler and easier to treat than leukemia.

The present study utilized the classic human leukemia HL-60 cell line, which is accepted as a valid model for testing anti-leukemic or general anti-tumor compounds. It can be induced to differentiate into mature neutrophil or macrophage/monocyte by some stimuli, depending on the inducing agents (12). These characteristics have also made HL-60 cells a useful model for studying genes whose expression can be correlated with cell differentiation. The results of our study support that gossypol plays a role in the control of proliferation of HL-60 cells. It demonstrated that gossypol in low concentrations (2-5 μM) induces differentiation in the leukemia cell line effectively, suggesting a possible use for gossypol in the differentiation treatment of leukemia.

## MATERIAL AND METHODS

### Materials and reagents

Gossypol, nitroblue tetrazolium (NBT), dimethyl sulfoxide (DMSO), TPA (12-O-tetradecanoylphorbol 13-acetate), Wright-Giemsa staining were purchased from Sigma (USA). Antibodies against total PBK (PDZ-binding kinase)/TOPK (T-LAKcell-originated protein kinase) (PBK/TOPK), phosphorylated PBK/TOPK, CD11b, CD13, CD14 and CD33 were purchased from BD Biosciences Pharmingen (USA). Fetal bovine serum (FBS) and RPMI-1640 were purchased from Gibco (USA).

### Cell culture

The classic human acute promyelocytic leukemia cell line HL-60 was obtained from American Type Culture Collection (ATCC) and maintained in RPMI1640 containing 10% heat-inactivated FBS, 2 μM L-glutamine, 100 U/ml penicillin, and 100 μg/ml streptomycin (Gibco, USA) in a humidified 5% CO_2_ atmosphere at 37°C. For experiments, cells were seeded at 5 × 10^4^ cells/ml in 10cm dishes and cultured for 96hr in the presence or absence of gossypol indicated.

### Cell proliferation and viability assay

Cell proliferation and viability were determined by Trypan blue exclusion test. HL-60 cells were seeded in the absence or in the presence of different concentrations of gossypol. Gossypol stock solution was made up to 12.5 mM with dimethyl sulfoxide and further diluted with fresh medium. Control flasks were treated with 0.2% DMSO alone. EC50 value (concentration of tested agent causing 50% inhibition of cell growth) was estimated after 72 hr exposure and only viable cells were counted. In the viability assay, the number of cells that did not take up Trypan blue was expressed as the percentage of the total cell number. All other experiments were performed at the concentrations of gossypol in which cell viability of >90% on 72 hr was observed (n=4 per experiment). For determination of growth rate, treatment at the selected concentrations was carried out up to 144 hr and aliquots were removed daily for determination of cell number. The medium was not changed during the induction period.

### Morphological changes

To determine changes in morphology, 2 × 10^4^ cells treated with gossypol in low concentrations (2-5 μM) for different periods were centrifuged (1000 rpm, 5 min, 20°C) onto a slide and then stained with Wright-Giemsa. The size and morphological changs were analyzed using a light microscope (Olympus, Japan).

### NBT reduction

To assess the effect of gossypol on the induction of cellular differentiation, exponentially growing HL-60 cells were treated with 2-5 μM gossypol for up to 96 hr. Next, the gossypol-induced myeloid differentiation was determined by using NBT reduction, in which the positive cells were proportional to the degree of differentiation into myeloid cells (13). In brief, after being washed with Hanks’ balanced salt solution (HBSS), cells were resuspended and incubated in 0.2 ml of HBSS containing 1 mg/ml NBT and 5 μg/ml TPA at 37°C for 30 min. The cells were then pelleted and fixed in 0.1 ml of 4% paraformaldehyde in PBS. Cells displaying intracellular black-blue formazan deposit were counted by microscopic examination. A minimum of 200 cells were examined in duplicate from four separate experiments.

### Cell cycle analysis

Effects of gossypol on the cell cycle were examined by DNA analysis using a Facscan flow cytometer (ELITE, Beckman-Coulter, USA) according to standard methods (14). Briefly, HL-60 cells were induced at a cell density of 8 × 10^5^ cells/ml in the presence of different concentrations of gossypol, then harvested, centrifuged, washed and resuspended in ice-cold 70% ethanol in PBS for 30 min. RNase A was added and samples were incubated for 30 min at 37°C. Propidium iodide (100 μl, 400 μg/ml, PI, Coulter, Cor. USA) was added and cytometric analysis was performed immediately. Red fluorescence was measured from 5 × 10^3^ cells/sample collected in histogram format using Lysis II software. Fluorescence intensity data were acquired using the Cell Quest Proprogram. The percentages of the analyzed cell population in G0/G1-, S-, or G2/M-phases was determined by the Mod Fit cell cycle analysis program.

### Surface marker analysis

Cells were stained with monoclonal antibodies (anti-CD11b, anti-CD13, anti-CD14 and anti-CD33) labeled with fluorescein isothiocyanate and phycoeritrine respectively. Cell cycle was analyzed by propidium iodide staining of ethanol-fixed, ribonuclease-treated cells followed by flow cytometry.

### Western blotting analysis (15)

HL-60 cells were treated with or without 5 μM gossypol for 96 hr, harvested, washed twice with ice-cold PBS and lysed in lysis buffer containing 50 mM Tris-HCl (pH7.4), 150 mM NaCl, 1%NP-40, 5 mM EDTA, 5 mM NaF, 2 mM Na_3_V_4_, 1 mM phenylmethyl sulfonylfluoride (PMSF), 5 μg/ml leupeptine, and 5 μg/ml aprotinin for immunoblotting of whole cell lysates. Lysates were then sonicated twice for 10 seconds, and supernatants were obtained by centrifugation (12,000 rpm, 4°C, 8 min). Lysates of whole cellular protein or cytosolic protein were boiled in SDS sample buffer (62.5 mM Tris-HCl, PH 6.8, 2% SDS, 10% glycerol, 50 mM DTT, and 0.1% bromophenol blue) for 5 to 10 min. Protein concentrations were detected by using a dye-binding protein assay kit (Bio-Bad, USA) under the guide of manufacturer’s manual. Equal amounts of lysate protein were run on 15% SDS-PAGE and electrophoretically transferred to PVDF membrane (Amersham–Pharmacia Biotech, USA). Blots were blocked with TBST buffer (500 mM NaCl, 20 mM Tris-HCl, PH 7.4, and 0.1% Tween 20) containing 5% nonfat dry milk and incubated (4°C, overnight) with specific primary antibodies above (anti-total PBK/TOPK, phosphorylated PBK/TOPK) in TBST buffer containing 5% bovine serum albumin. Blots were further incubated for 1hr with horseradish peroxidase (HRP)-conjugated secondary antibodies. Bound antibodies were detected by reaction with 3,3’-diaminobenzidine (DAB).

### Statistical analysis

Statistical analysis was performed using Statistical Program for Social Sciences (SPSS) 10.0 Soft ware. Differences between treated and control data were analyzed by *t* test. In all cases, *p*<0.05 was considered to be significant.

## RESULTS

### Subtoxic concentrations of gossypol reduces growth rate of HL-60 cells

EC_50_ value was found to be 10.2 μM and selected concentrations (2-5 μM) were used in all subsequent experiments. A statistically significant duration-dependent reduction of the cell proliferative capacity was observed in HL-60 cells cultured with subtoxic concentrations of gossypol continuously for 120 hr. Mean results from four independent experiments are shown. The highest cytostatic response (reduction of the proliferation rate to about 20% of control on 72 hr) was seen in cell cultures exposed to 6 μM gossypol. We selected some concentrations in this scope (2-5 μM) of gossypol to detect the differentiation effects in HL-60 cells.

### Gossypol affects HL-60 cells morphology

**Figure 1:**
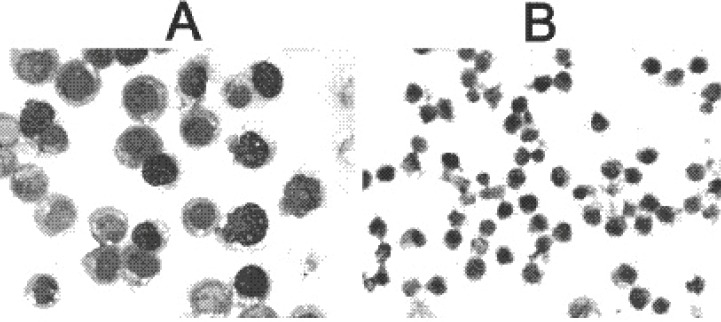
The size (×400) and morphological changs between the control and treated HL-60 cells (Group A: Control, Group B: Treated cells).

After 96 hr of culture, the average cell size of the HL-60 cells treated with gossypol was much smaller than that of control cells (data not shown). The most pronounced cell size change measured by forward scatter was visible in cultures treated with gossypol. When more mature morphological changes were observed by Wright-Giemsa staining, untreated HL-60 cells were predominantly promyelocytes containing a large nucleus and granules in cytoplasm. On the other hand, differentiated myeloid cells including myelocyte, metamyelocyte, banded and segmented neutrophils were observed in the treated HL-60 cells for 96 hr. The cells treated with gossypol showed the granulocytic morphologic maturation with increased cytoplasm and polygonal nuclei. The nuclei became condensed, shrunk, twisted and lobulated, and the nuclei versus cytoplasmic ratio decreased, suggesting the process of cell differentiation occurred (Fig 1).

### Gossypol elevates NBT reduction positive rate

To assess myeloid differentiation in HL-60 cells, we used NBT reduction assay. In untreated HL-60 cells, the percentage of NBT-positive cells was very low (less than 5%). The data plotted in Table 1 show that myeloid differentiation was already detectable after 48hr of treatment with gossypol reaching 20% of NBT-positive cells. The differentiation on 96 hr was accompanied with a marked inhibition of cell proliferation but no cytotoxicity. The percentage of NBT-positive cells increased as the gossypol incubation period was prolonged. More than 90% of the cells were NBT-positive on 96 hr (Table 1).

**Table 1:** NBT reduction in gossypol-treated human leukemia HL-60 cells (in percentage)

	48 hr		96 hr	
	2 μM	5 μM	2 μM	5 μM

HL-60	3.1 ± 1.4	4.3 ± 1.2	5.0 ± 0.8	3.5 ± 2.0
Treated HL-60	19.5 ± 3.5^a^	46.2 ± 4.1^a^	51.2 ± 3.8^a^	86.4 ± 2.6^a^

a Values are presented as mean ± S.D. (*n* = 4). Significant differences between values for control and for various concentrations of gossypol are shown (*P* < 0.001).

### Induction of differentiation of HL-60 cells is accompanied by cell-cycle arrest

To further characterize the effect of gossypol, which induced HL-60 cells differentiation, cell cycle analysis was performed on propidium iodide-stained cells. Flow cytometric analysis of DNA content revealed that in untreated control HL-60 cell cultures, G0/G1-, S-, and G2/M-phase cells represented approximately, 42, 38, and 20% of the total cell population, respectively. Exposure of exponentially growing cultures to gossypol at concentrations which did not affect cell viability resulted in a marked increase in cell number in G0/G1-phase. Decrease in the number of cells in S-, and G2/M-phase in the cells treated with gossypol (Table 2). A marked loss of cells in these phases correlated with an accumulation of the HL-60 cells in G0/G1-phase.

**Table 2:** Distribution of cell cycle in gossypol treated HL-60 cells (in percentage)

	G0/G1	S	G2/M	Sub-G1

HL-60	42 ± 1.5	38 ± 3.4	20 ± 3.0	0 ± 0
Treated HL-60	65 ± 3.0^a^	17 ± 3.4^a^	15 ± 1.6	3 ± 0.5

a Value significantly different from those of control cells (*P*<0.01). The percentage of cell number in each cell phase is presented as mean ± S.D. (*n*=4).

### Gossypol changes surface markers

CD13 (FITC-labelled) expression was used as a maker of granulocytic differentiation, while CD14 (PE-labelled) expression was used to monitor monocytic differentiation. In comparison with the untreated cells, the amounts of CD13 and CD14 positive cells were increased significantly after 96 hr treatment with 4 μM of gossypol (*P*<0.01, n=4). On the other hand, to CD11b and CD33, no obvious changes were observed (*P*>0.05, n=4) (Table 3).

**Table 3:** Changes of surface markers in gossypol-treated HL-60 cells (in percentage)

	CD11b	CD13	CD14	CD33

HL-60	0.7 ± 0.2	7.9 ± 1.5	10.5 ± 1.2	99.0 ± 0.6
Treated HL-60	2.6 ± 0.1	56.5 ± 2.2^a^	31.4 ± 0.9^a^	99.7 ± 0.8

a Value significantly different from those of control cells (*P*<0.01). The percentage of cell number in each cell phase is presented as mean ± S.D. (*n*=4).

### Gossypol modulates PBK/TOPK pathway in HL-60 cells

**Figure 2:**
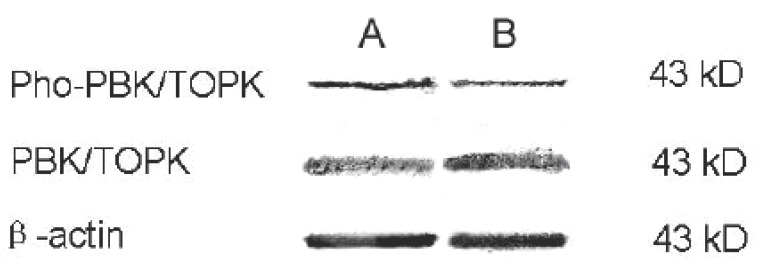
Effect of gossypol on the expression of total PBK/TOPK and phosphorylated PBK/TOPK in HL-60 cells (Group A: Control, Group B: Treated cells). Treated HL-60 cells did not demonstrate a significant difference in expression of total PBK/TOPK compared to control cells. To phosphorylated PBK/TOPK, the change is obvious. As a negative control protein, β-actin also demonstrated no change.

We investigated the possible involvement of the PBK/TOPK pathway in the gossypol-induced cellular differentiation of HL-60 cells. It was found that there was no obvious change in total PBK/TOPK, but to active phosphorylated PBK/TOPK, it decreased prominently. No changes were seen in β-actin, used as a control (Fig 2). These results demonstrated that a PBK/TOPK-dependent signaling pathway was involved in gossypol-induced differentiation in HL-60 cells.

Due to low number of women participating in the bone density observation test, results were expressed as groups’ means and standard deviation only, since calculation of statistically-significant differences would lack credibility. Observed increase in bone density markers due to Maca-GO administration was accompanied by a substantial decrease in FSH (55%) and an increase in E2 values (135%), while in Placebo group, during the same length of the Trial, there was much reduced change observed in the same hormone values (11% decrease for FSH and 83% increase in E2).

### Kupperman’s Menopausal Index (KMI) and Greene’s Menopausal Score (GMS)

The objective in analysis of menopausal symptoms was to examine the effect of Maca-GO treatment administered over variable time frames (one or two months) intermittently with Placebo (at pre and post Maca-GO administration), on the alleviation of menopausal symptoms as subjectively assessed by early-postmenopausal women during personal interviews with gynecologists. In analysis of the answers provided by participants to the questionnaires according to Kupperman and Greene, the emphasis was put on those symptoms which had been indicated by women at admission as having the most pronounced effects on their life as they entered menopause Listed in descending order of priority, the following symptoms reflect the degree of discomfort attributed to their early-postmenopausal stages: hot flushes, nervousness, excessive sweating (profuse perspiration), interrupted sleep pattern, depression, general weakness, headache, joint pain, heart palpitations, loss of body balance and numbness in hands and/or legs-feet.

## DISCUSSION

As we know, leukemia is characterized by a break-down in neoplastic cell maturation. To restore the normal differentiation, such patients are treated with differentiation therapy using differentiating agents like alltrans retinoic acid (ATRA) (16). However, it is also found that the rapid catabolism of retinoic acid makes it very difficult to maintain the effective plasma levels of ATRA (17) and remission can not be reached in all cases and the therapy has side effects, such as drug resistance and hypercalcemia (18). Therefore, it seems very worthwhile to search for other substances which induce differentiation alone or in combination with established inducers of differentiation (19).

In the present study, gossypol was found to inhibit the proliferation of human promyelocytic leukemia cell line HL-60 and induce the cells to differentiate toward granulocytes from the following evidence.

- Gossypol inhibited HL-60 cells proliferation and increased NBT reduction activity.
- Gossypol caused morphological changes toward granulocyte-like cells after culture for 4 days; indented nuclei and banded neutrophils were observed in gossypol-treated cells.
- Gossypol-treated cells were arrested mainly at G0/G1 phase.
- Gossypol induced the increase in the expression of cell surface antigen CD13 and CD14.
- Gossypol modulated PBK/TOPK pathway in the differentiated HL-60 cells.

However, the detailed mechanism for the induction of differentiation by gossypol is unknown, and it remains unclear whether gossypol effectively induces the elimination of malignant cells via the differentiation in vivo.

Induction of leukemia cells differentiation occurs at low concentrations of drugs, which is not toxic for the cells. Therefore, this less toxic approach, referred to as differentiation therapy which involves compounds to modify the state of differentiation and growth of neoplastic cells, may provide a support to cytotoxic chemotherapy and radiotherapy (20). It had been found that gossypol could induce apoptosis in some solid tumor and leukemia cell lines (7), but it has not been used to treat leukemia yet. In the present study, we found the continuous exposure of the cells to nanomolar, subtoxic concentrations of gossypol could stimulate differentiation. Our results provided evidence that there was a correlation between the degree of inhibition of proliferation and accumulation of myeloid cells. At the same time, the results showed that induction of differentiation was dose- and time-dependent. The correlation between inhibition of proliferation and induction of differentiation was significant. In this respect, the chemical induction of differentiation is similar to normal hematopoiesis, where the proliferative capacity of cells decreases as the degree of differentiation increases.

PBK/TOPK is a serine/threonine kinase that is phosphorylated and active during mitosis (21). It is suggested that it may be important for the growth and differentiation of malignant cells particularly of the hematopoietic origin. First, sequences corresponding to this serine/threonine kinase were identified by differential mRNA display to be up-regulated in Burkitt’s lymphoma cells as compared to hyperplastic tonsillar B cells (22). Second, PBK/TOPK was cloned on the basis of an *in vitro* interaction with the PDZ protein binding domain of the human homolog of the Drosophila tumor suppressor Discs-large (hDlg) (21). Third, expression of PBK/TOPK was found to be up-regulated in a variety of neoplastic cell lines and in some clinical samples from patients with leukemia, myeloma (23). Increased PBK/TOPK expression has been observed in highly proliferative malignant cell lines, and PBK/TOPK expression is down-regulated during terminal differentiation of leukemia cells. TPA-induced differentiation and growth arrest of HL-60 leukemia cells led to profound down-regulation of PBK/TOPK. Potential substrates of PBK/TOPK include p38 MAPK and c-Myc (23, 24). In this paper, additional evidence is presented to support the role of PBK/TOPK in the differentiation of leukemia HL-60 cells; it indicates that gossypol can induce differentiation in leukemia cells (24). At the same time, further studies are needed to fully elucidate the detailed mechanism of action of gossypol-induced differentiation in HL-60 cells.

In conclusion, we have shown that efficiency of myeloid differentiation of HL-60 cells was dose- and time-dependent. This study is the first to identify gossypol’s pro-differentiated effects on leukemia cells. These results also suggest that gossypol induces differentiation through a PBK/TOPK down-regulation pathway. At subcytotoxic concentration, gossypol has the greatest potential to inhibit proliferation and to induce differentiation in vitro. Differentiation is preceded by cell-cycle arrest in G0/G1-phase. The concentrations of gossypol used in this study are pharmacologically relevant. Therefore, when used at low, nanomolar concentrations, gossypol was not cytotoxic but was able to induce differentiation of rapidly dividing cells. So it might be exploited as potential therapeutic agents, chemoprevention or chemotherapeutic adjuvant especially in combination drug for leukemia therapy.
